# Targeted sequencing of the *BDNF* gene in young Chinese Han people with major depressive disorder

**DOI:** 10.1002/mgg3.1484

**Published:** 2020-08-31

**Authors:** Chenyu Zhang, Liuyi Ran, Ming Ai, Wo Wang, Jianmei Chen, Tong Wu, Wei Liu, Jiajia Jin, Suya Wang, Li Kuang

**Affiliations:** ^1^ Department of Psychiatry The First Affiliated Hospital of Chongqing Medical University Chongqing China; ^2^ Mental Health Center University‐Town Hospital of Chongqing Medical University Chongqing China

**Keywords:** brain‐derived neurotrophic factor, major depressive disorder, next‐generation sequencing

## Abstract

**Background:**

Adolescence and young adulthood are considered the peak age for the emergence of many psychiatric disorders, in particular major depressive disorder (MDD). Previous research has shown substantial heritability for MDD. In addition, the brain‐derived neurotrophic factor (*BDNF*) gene is known to be associated with MDD. However, there has been no study conducting targeted sequencing of the *BDNF* gene in young MDD patients so far.

**Method:**

To examine whether the *BDNF* gene is associated with the occurrence of MDD in young patients, we used targeted sequencing to detect the BDNF gene variants in 259 young Chinese Han people (105 MDD patients and 154 healthy subjects).

**Results:**

The *BDNF* variant rs4030470 was associated with MDD in young Chinese Han people (uncorrected *p* = 0.046), but this was no longer significant after applying FDR correction (*p* = 0.552, after FDR correction). We did not find any significant differences in genotype or haplotype frequencies between the case and control groups, and furthermore discovered no rare mutation variants any of the 259 subjects.

**Conclusion:**

Our results do not support an association of the *BDNF* gene variants with MDD in young people in the Chinese Han population.

## Graphical abstract

We conducted a targeted sequencing to detect BDNF gene variants in 259 young Chinese Han people (105 MDD patients and 154 healthy subjects). However, we dd not find any significant differences in allele、genotype or haplotype frequencies between case and control groups, and furthermore discovered no rare mutation variants any of the 259 subjects. Our results do not support an association of BDNF gene variants with MDD in young people in the Chinese Han population.

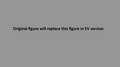

## 1 INTRODUCTION

Major depressive disorder (MDD) is one of the common psychiatric conditions, with its peak in adolescence and young adulthood (Ronald C et al., 2007). It is also the leading cause of disability among young people around the world (Michael & Charles, 2012). The disease is not only a major risk factor for suicide (Hawton & van Heeringen, 2009), but also causes serious social and educational problems, for example, by increasing the chances of dropping out (Fletcher, 2009; Lewinsohn, Rohde, & Seeley, 1998). Because of clinical heterogeneity and multifactorial etiology, the pathogenesis of the disease and its underlying factors have been difficult to understand. At present, however, it can be confirmed that genetic factors play a role in the occurrence of MDD, with a heritability of about 30%–50% (Bierut et al., 1999). Some studies have defined MDD with an onset‐age below 30 as early onset MDD, as it has a higher average heritability than MDD with an age of onset above 30 years (Cheng et al., 2014; Lyons et al., 1998; Tozzi et al., 2008; Yang et al., 2011). Therefore, the early onset MDD may represent a stronger genetic MDD subtype.

Recently, the neurotrophic hypothesis of depression has attracted much attention, and the gene encoding for brain‐derived neurotrophic factor (*BDNF*) (OMIM: 113505) has become one of the most interesting candidate genes for MDD heritability and pathogenesis. The *BDNF* gene is located on human chromosome 11p14, containing 11 exons and 9 promoters (Maisonpierre et al., 1990). *BDNF* plays a crucial role in nerve development, promoting proliferation, differentiation, maturation, and survival of nerve cells, and participates in the regulation of synaptic plasticity and cognitive function (Huang & Reichardt, 2001; Wu, Molteni, Ying, & Gomez‐Pinilla, 2003).

Previous studies have found that neurodevelopmental factors are likely to be involved in pathogenesis when depression is developed at an early age (Rice et al., 2019). Although genome‐wide association studies have identified a few common variants associated with MDD, the function of these common variants is generally unclear, the effects are weak (Cai et al., 2015; Hyde et al., 2016), and the proportion of common variants can only explain 21% of the genetic risk for depression (Cross‐Disorder Group of the Psychiatric Genomics Consortium, et al., 2013), indicating a partial “loss” of heritability (Manolio et al., 2009). However, with the development of next‐generation sequencing techniques, many studies have found that rare mutations in genes can explain some of this “loss of heritability” (Amin, Belonogova, et al., 2016; Amin, Jovanova, et al., 2016; Amin et al., 2017; Pirooznia et al., 2015). In addition, several studies have suggested that these rare gene variants may be associated with depression (Amin et al., 2017; Pirooznia et al., 2015; Yu, Arcos‐Burgos, et al., 2018). As a result, both common and rare variants may increase the risk of developing the depression (Wong et al., 2016; Yu, Arcos‐Burgos, et al., 2018).

Based on the important functions of the *BDNF* gene in neurodevelopment and cognitive function, we hypothesized that rare genetic variations of the *BDNF* gene might be related to MDD in young people. While several studies have looked into the relationship between the *BDNF* gene and MDD in young people, no study has so far investigated the association of *BDNF* sequence variants in early onset depression. In this study, we explored the relationship between common and rare functional variation in the *BDNF* gene in young people with depression as an early onset subtype of depression using next‐generation sequencing.

## 2 MATERIALS AND METHODS

### 2.1 Ethical compliance

The study obtained approval by the ethical review board of The First Affiliated Hospital of Chongqing Medical University (No. 2017‐157). All subjects provided written informed consent. If participants were aged below 18 years, their parents or legal guardians were required to sign the informed consent form on their behalf.

### 2.2 Participants

Our study comprised a total of 259 participants. We recruited 105 patients from the Department of Psychiatry at the first affiliated hospital of Chongqing Medical University and the Mental Health Center at the University‐town hospital of Chongqing Medical University in Chongqing. Participants included individuals with MDD based on criteria of the Diagnostic and Statistical Manual of Mental Disorders, IVth edition (DSM‐IV‐TR) (*n* = 105) and healthy control participants (*n* = 154).

All participants were of Chinese Han descent and were diagnosed with MDD according to the DSM‐IV criteria but not suffering from other mental illnesses. Individuals with severe physical illnesses, mental retardation, or a history of alcohol, or psychoactive substance abuse were excluded from this study. The 154 control individuals were healthy volunteers that were recruited from the local school, without history of mental disease or physical or neurological illness.

In this study, we chose early onset depression as the extreme phenotype. Extreme phenotype sampling is a strategy which selecting individuals from the extremes of the trait distribution (such as age of onset) (Li et al., 2019; Manolio et al., 2009), and it can increase the ability and statistical power to explore rare variants. The demographic information of participants was as follows: we included 105 MDD patients (further abbreviated as MDD), including 73 females and 32 males, which were all aged between 13 and 25 years and had a mean age of 18.18 ± 2.741 years. We included 154 controls (further abbreviated as CON), including 92 females and 62 males, all aged between 14 and 24. The mean age of the control group was 18.81 ± 1.759 years. The statistical results about gender and age between the MDD and the control group are as follows: *χ* ^2^ = 2.585, *p* = 0.108 and *t* = −2.083, *p* = 0.039.

### 2.3 DNA sampling

We collected 2–5 ml of peripheral venous blood from all subjects in a DNA extraction EDTA anticoagulant tube. Genomic DNA was extracted using the TIANamp blood genomic DNA kit (Tiangen Biotech (Beijing) Co., Ltd), and genomic DNA quality and quantity were detected using a Nanodrop 2000 spectrophotometer (NanoDrop Technologies, Wilmington, DE, USA). For all samples, the concentration was ≥20 ng/μl. The genomic DNA was stored at −80℃ and sent to Genesky Biotechnologies Inc., Shanghai for FastTargetTM target region sequencing.

### 2.4 Targeted sequencing of *BDNF*

The target region included all coding and regulatory regions of the *BDNF* gene (NG_011794.1): the regulatory region included the 500 bp promoter region, the 15 bp exon‐intron boundary region, the 5′ untranslated region (5′ UTR), and the 3′ untranslated region (3′ UTR). We used the primer 3 software to design specific primers for gene target regions. The AB 2720 type PCR thermal cycler (Applied Biosystems, Waltham, MA, USA) was used for the amplification reaction. All PCR products were sequenced using the 2 × 150 bp paired‐end sequencing mode on an Illumina HiSeq platform (Illumina Inc, San Diego, CA, USA).

### 2.5 Bioinformatics analysis

We used FastQC (http://www.bioinformatics.babraham.ac.uk/projects/fastqc/) to evaluate the quality of the raw sequencing data. The BWA v0.7.15‐r1140 software (http://bio‐bwa.sourceforge.net/) was used to align the raw sequencing data to the human genome reference sequence (UCSC hg19), and the preliminary alignment results were obtained in BAM format. The preliminary comparison result of BWA software was corrected using the GATK standard software (https://software.broadinstitute.org/gatk/best‐practices/), which mainly included local realignment and base quality recalibration. Using both the VarScan v2.3.9 software (http://varscan.sourceforge.net/) and GATK HaplotypeCaller module (https://software.broadinstitute.org/gatk/), we identified the mutation sites. The mutation sites were compared with a known population database, a functional database and a disease database with the ANNOVAR package (http://annovar.openbioinformatics.org/en/latest/) to obtain the most biologically significant variation sites and carry out the functional annotation.

### 2.6 Statistical analyses

Age and gender matching were statistically analyzed using an adopted independent sample *t*‐test and chi‐square test, respectively, in SPSS 25.0. The single nucleotide polymorphisms (SNPs) were analyzed using the PLINK 1.07 software (http://pngu.mgh.harvard.edu/purcell/plink/) and the Hardy–Weinberg genetic equilibrium test was performed using a chi‐square test for genotype distribution between the two groups. The genotype and allele frequency between the two groups were tested using a chi‐square test, and Odds ratios (OR) and 95% confidence intervals (CI) were calculated. The linkage disequilibrium (LD) of the SNPs the gene was analyzed with the Haploview software and D’ value was used as a measure. A higher D’ value indicated a higher degree of LD, and we obtained haplotypes with strong correlations. The haplotype frequency comparison between the two groups was based on a chi‐square test, and the correlation between haplotype and the disease was analyzed using OR and the 95% CI. The correlation analysis of single SNPs was assessed with a false discovery rate (FDR) applied for the correction test. SNP‐SNP interaction analyses were performed using the generalized multifactor dimensionality reduction (GMDR) software (http://www.ssg.uab. edu/gmdr/) (Lou et al., 2007). Two‐tailed *p*‐values <0.05 were considered statistically significant.

## 3 RESULTS

### 3.1 Sequencing data

The average sequencing depth was 572X, and the proportion of samples with an average effective sequencing depth (>200X) was 97%. All variations were classified into common variants (MAF ≥1%) and rare variants (MAF <1%) based on the minor allele frequency of variations in the control group. In total, we found 10 SNPs and 1 short tandem repeat (STR) in the target region of *BDNF* gene, all of which were common variations, as shown in Table S1. Our subsequent analysis focused on these SNPs. We did not detect any rare variations in either patient or control group. The genotype distribution of all variation sites was in accordance with the Hardy–Weinberg genetic equilibrium.

### 3.2 Single marker association analysis

As shown in Table 1, the genotype distribution of the 10 polymorphisms of the *BDNF* gene showed no differences between young MDD patients and controls. Of all markers, only the rs4030470 allele showed an association with young MDD patients, with an uncorrected *p* of 0.046. However, this association did not remain significant after FDR correction (*p* = 0.552 after FDR correction).

**Table 1:** Genotype and allele distributions of *BDNF* gene polymorphisms in young people (patients with major depressive disorder or controls)

SNP‐ID	Case (%)			Control (%)			*χ* ^2^	*p*‐value	MAF (%)		*χ* ^2^	*p*‐value	OR (95% CI)
	M/M	M/m	m/m	M/M	M/m	m/m			Case	Control			
rs7124442	86.7	12.4	0.9	83.1	16.2	0.7	0.798	0.670	7.1	8.7	0.441	0.506	0.800 (0.415–1.544)
rs11030099	31.7	50.0	18.3	33.1	48.7	18.2	0.058	0.971	43.2	42.5	0.027	0.868	1.031 (0.722–1.47)
rs79642557	83.7	15.4	0.9	81.2	17.5	1.3	0.279	0.869	8.6	10.0	0.287	0.591	0.846 (0.460–1.557)
rs6265	26.7	54.3	19.0	31.2	48.1	20.7	1.004	0.605	46.1	44.8	0.096	0.755	1.057 (0.743–1.504)
rs11030101	50.4	38.1	11.5	50.0	42.2	7.8	1.154	0.561	30.4	28.9	0.149	0.698	1.079 (0.735–1.583)
rs200712840	81.9	18.1	0	78.6	21.4	0	0.432	0.51	9.0	10.7	0.384	0.535	0.829 (0.457–1.501)
rs202011320	84.8	15.2	0	78.6	20.1	1.3	2.482	0.289	7.6	11.3	1.973	0.160	0.643 (0.346–1.195)
rs4030470	71.4	22.9	5.7	79.9	18.2	1.9	3.81	0.148	17.1	11.0	3.981	0.046*	1.667 (1.005–2.765)
rs2883187	26.7	53.3	20	30.5	48.1	21.4	0.728	0.694	46.6	45.4	0.073	0.785	1.05 (0.738–1.493)
rs71050932	27.6	52.4	20	29.9	48.7	21.4	0.338	0.844	46.1	45.7	0.008	0.926	1.017 (0.715–1.446)

Note

Case, young patients with major depressive disorder; CI, confidence interval; Control, healthy young people; M, major allele; m, minor allele; MAF, minor allele frequency; OR, odds ratio.

BDNF gene is based on NCBI Reference Sequence NG_011794.1.

* After FDR, the adjusted *p*‐value was 0.552.

### 3.3 Haplotype association analysis

**Figure 1:**
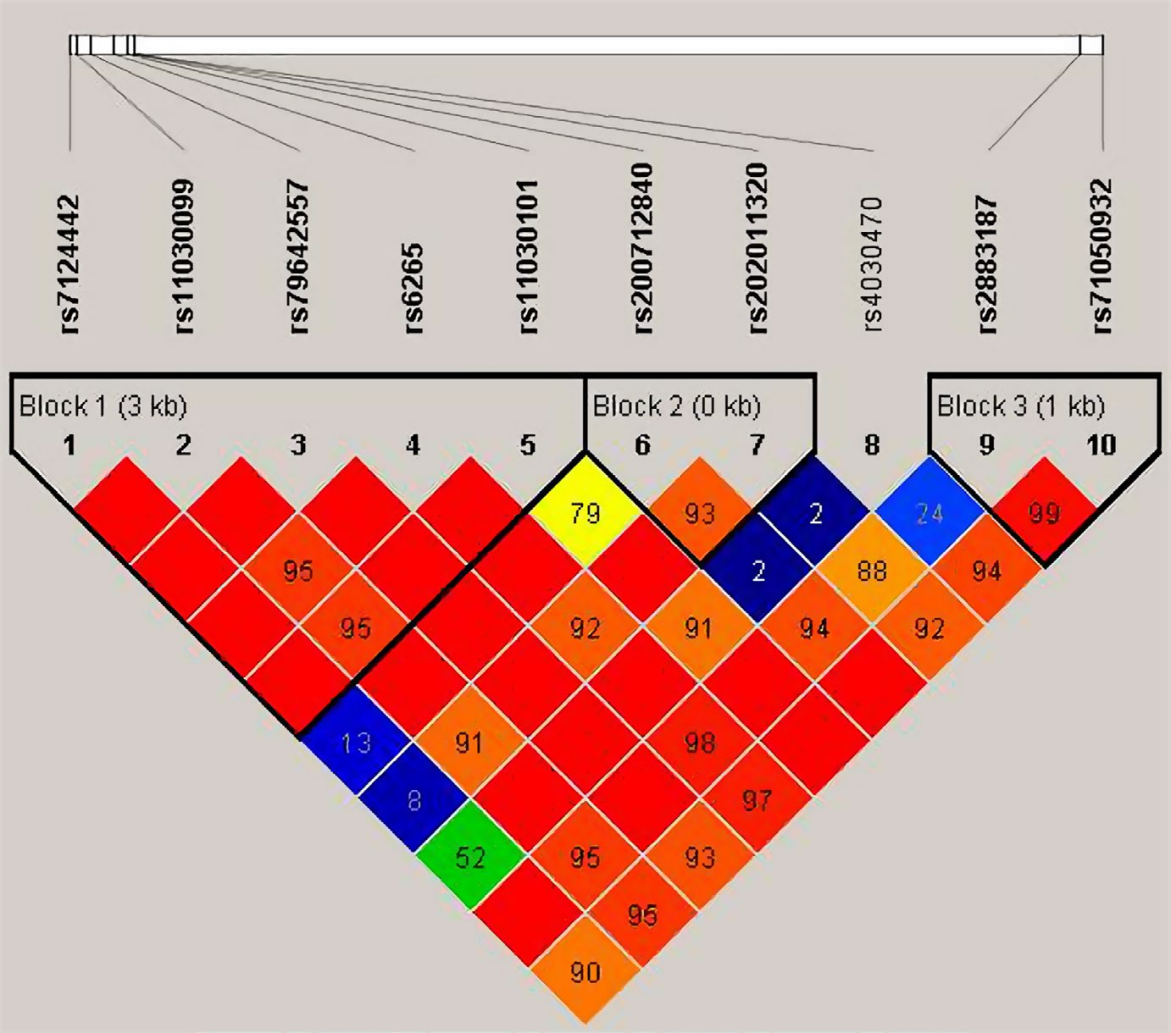
LD estimates across the *BDNF* gene (NCBI Reference Sequence: NG_011794.1) in the analyzed groups. We found three haplotype blocks in 10 SNPs in *BDNF* gene. A higher intensity of red indicates a higher degrees of LD

Linkage disequilibrium (LD) analysis of the 10 functional polymorphism loci in the *BDNF* gene region identified three haplotype blocks with strong correlation (Figure 1). Block 1 consisted of five SNPs (rs7124442, rs11030099, rs79642557, rs6265, and rs11030101), Block 2 consisted of two SNPs (rs200712840 and rs202011320), and Block 3 consisted of two SNPs (rs2883187 and rs71050932). We conducted an association analysis of *BDNF* haplotypes with MDD in young patients. The results are demonstrated in Table 2. None of the *BDNF* gene haplotypes were associated with MDD in young people in our study.

**Table 2:** Differences in *BDNF* gene haplotypes between young patients with major depressive disorder and controls

Haplotype	Case (%)	Control (%)	*p*‐value	OR (95% CI)
Block 1				
CCGCA	15 (10.7)	27 (14.2)	0.339	0.715 (0.359–1.421)
TAATA	20 (14.0)	30 (15.0)	0.814	0.930 (0.506–1.706)
TCGCA	34 (22.3)	51 (22.5)	0.961	0.986 (0.580–1.677)
TCGCT	63 (35.0)	88 (33.8)	0.793	1.057 (0.696–1.603)
TCGTA	6 (4.5)	11 (6.5)	0.444	0.663 (0.231–1.899)
Block 2				
—	15 (7.1)	33 (10.7)	0.148	0.611 (0.313–1.192)
Block 3				
G‐	16 (9.5)	27 (11.1)	0.593	0.836 (0.433–1.613)
GCATTT	96 (46.1)	141 (46.0)	0.986	1.003 (0.701–1.434)

Note

Block 1: rs7124442‐rs11030099‐rs79642557‐rs6265‐rs11030101; Block 2: rs200712840‐rs202011320; Block 3: rs2883187‐rs71050932; Case: young patients with major depressive disorder; Control: healthy young people.

### 3.4 SNP‐SNP interactions

To explore the epistatic gene interaction, we used a GMDR approach to evaluate the SNP‐SNP interactions between the 10 SNPs in the *BDNF* gene. There were no significant interaction effects between any of the seven SNP‐SNP interactions in the GMDR model of the young MDD patient group (Table S2).

## 4 DISCUSSION

In this study, we conducted targeted sequencing of all *BDNF* exons and their flanking regions in a total of 259 young Chinese Han people, consisting of 105 MDD patients and 154 healthy controls. Our results showed that the rs4030470 allele was associated with MDD in young patients before correction (*p* = 0.046), but this association did not remain statistically significant after FDR correction (*p* = 0.552). Moreover, we found no statistical difference in genotype or haplotype frequencies between patients and controls, and no rare mutation variants were found in any of the participants. Furthermore, in the current study, we identified no interactions between the 10 SNPs of the *BDNF* gene.

*BDNF* encodes a neurotrophic protein which is highly expressed in central nervous system, particularly in the hippocampus and cortical neurons (Beck, 1994). During development and in adulthood, *BDNF* plays a significant role in neural development (Huang & Reichardt, 2001; Schinder & Poo, 2001). Here, we conducted a study in adolescents, which are in an important period of neurodevelopment (Keshavan, Giedd, Lau, Lewis, & Paus, 2014). Based on the literature, it appears that MDD in young people is related to alterations in neurodevelopment (Hagan et al., 2015; Paus, Keshavan, & Giedd, 2008; Truong et al., 2013). In addition, studies have shown that *BDNF* is associated with the development of neurodevelopmental disorders, such as schizophrenia (Zhang et al., 2016) and Attention‐Deficit/Hyperactivity Disorder (ADHD) (Liu et al., 2014; Zhang et al., 2018).

The rs6265 (Val66Met) variant of the *BDNF* gene lies in the functional coding region and has received much attention. Aldoghachi et al. (2019) and Ribeiro et al. (2007) found that the rs6265 polymorphism of *BDNF* gene is associated with MDD. Youssef et al. (2018) found lower levels of BDNF in the anterior cingulate cortex and caudal brainstem in MDD patients compared with nondepressed subjects, and their results also showed that the rs6265 variant was associated with MDD. Moreover, Cruz‐Fuentes et al. (2014) did not find a relationship between the rs6265 polymorphism of *BDNF* gene and MDD in Mexican youth population, which is consistent with the findings of our study. Similarly, a meta‐analysis by Gyekis et al. (2013) identified no association of the *BDNF* rs6265 polymorphism with MDD. The reason for the apparent inconsistency of these research findings may be due to a difference in genetic backgrounds between populations. In addition, different study inclusion criteria may account for a part of the discrepancy, as the study by Cruz‐Fuentes et al. (2014) and our study were about young people, while other studies were carried out in the adult population. Differences in age may furthermore be a factor explaining the discrepancy among results of these studies.

In addition to the rs6265 polymorphism, there have also been other polymorphisms whose association with MDD were studied. Domschke et al. (2009) and Musil et al.(2013) found that the rs7124442 variant of the *BDNF* gene was not associated with MDD. Similarly, our present study did not identify and association of the *BDNF* rs7124442 variant with MDD either. While there are no positive reports of the rs7124442 related to MDD, Zhang et al.(2010) found gene‐gene interaction effects between *BDNF* and *GSK3B* in mood disorders in a Chinese population, which include the association of the *BDNF* rs7124442 variant and Glycogen synthase kinase 3 beta (*GSK3B*) rs6782799, *BDNF* rs6265, *BDNF* rs7124442, and *GSK3B* rs6782799.

For most common human diseases, common mutations typically only explain a small proportion of heritability of the disease, while rare mutations explain part of remaining (Manolio et al., 2009). For example, rare structural variants of affected genes have been shown to influence epilepsy, mental retardation, autism, and schizophrenia (Stankiewicz & Lupski, 2010). Interestingly, Licinio, Dong and Wong (2009) found several rare variants of the *BDNF* gene in a case‐control study (272 MDD patients and 264 healthy controls) in Mexican Americans. Regretfully, they did not further analyze the relationship between these rare variants and MDD. In our current study, we did not identify any rare variants. An explanation for this discrepancy may be the different genetic background. In addition, the pathogenesis of MDD is very complex and includes a high genetic heterogeneity (Kupfer, Frank, & Phillips, 2012).

Studies have reported that the risk for MDD was also affected by other types of genetic variants, such as copy number variation (CNV) and STR (Yu, Baune, Wong, & Licinio, 2017, 2018). Interestingly, we did find an STR variation (rs3838785) in our current study, but there was no difference in genotype frequency and allele frequency between controls and cases (see Table S3). However, Yu, Baune, et al. (2018) reported that STRs were more common in healthy controls than MDD cases in a Mexican‐American population, but this result was not replicated in a study sampling Australians with European ancestry. Additionally, via whole genome‐sequencing, Yu, Baune et al. (2017) found that deletion CNVs were more frequent in MDD patients than healthy controls in the Mexican‐American sample. Zhang et al. (2019) conducted a meta‐analysis which found that rare CNVs may increase the risk for MDD. However, our study was unfortunately not sensitive enough to detect CNVs as the target sequences were evaluated based on an amplification strategy. In future studies, we will, therefore, further explore the structural mutations related to MDD risk using whole‐genome sequencing or microarrays.

To overcome the shortcomings of the small sample size of our study, we used a one‐sided extreme phenotype sampling method, as we aimed to focus on early onset MDD which is a strong genetic MDD subtype (Lyons et al., 1998; Wallace, Chapman, & Clayton, 2006; Yang et al., 2011). Using targeted sequencing on individuals with extreme or unusual phenotypes is, therefore, an effective strategy to research rare and low frequency variants and structural variants (Manolio et al., 2009). Studies have previously suggested that early onset MDD is genetically similar to schizophrenia and bipolar disorder (Power et al., 2017). To some extent, early onset depression can be considered as a subtype of depression. Therefore, in our study, we conducted extreme phenotypic sampling by assessing early onset depression (Manolio et al., 2009). Moreover, in one of our previous studies, using extreme traits sampling, we found that rare variants of serotonin transporter gene (*SLC6A4*) may contribute to the occurrence of MDD with suicide ideation in young people (Ran et al., 2020).

To the best of our knowledge, this is the first study to carry out targeted sequencing of the *BDNF* gene in young people. Targeted sequencing is a next‐generation sequencing approach which has previously been used to find rare mutations. Unfortunately, we did not detect any rare mutations in current study. However, although we did not identify any rare mutations in more than 200 people, this study can provide value for future sequencing studies of the *BDNF* gene.

Our study does have some limitations which should be considered when interpreting our findings. First, we cannot completely exclude that a lack of statistical power affected our results. Although we did use extreme phenotypic sampling to improve our statistical power, our sample size is small. Therefore, replication studies with larger sample sizes will be necessary to confirm our results. Additionally, previous studies have investigated interactions of the *BDNF* gene with environmental exposure in pathogenesis of MDD in young people (Youssef et al., 2018). In our study, we did not conduct any analysis of environmental factors. Finally, we only investigated the association in *BDNF* gene with the psychopathology of depression. However, as is well known, there are several other neuropeptides which may be associated with MDD, such as glial‐derived neurotrophic factor (*GDNF*) (Tsybko, Ilchibaeva, & Popova, 2017) and nerve growth factor (*NGF*) (Wiener et al., 2015).

## 5 CONCLUSION

To conclude, in our study of MDD in young Chinese Han people, we did not identify a significant association between the pathology of MDD and *BDNF* gene variants. These results indicate that the *BDNF* gene may not be involved in the pathogenesis of MDD in young Chinese people. However, further targeted sequencing studies with larger sample sizes will be necessary to determine whether genetic variation in *BDNF* influences the occurrence of MDD.

## CONFLICT OF INTEREST

Authors have no conflict of interest to declare.

## AUTHORS CONTRIBUTIONS

Chenyu Zhang and Liuyi Ran analyzed the data and wrote the paper. Ming Ai, Wo Wang, and Jianmei Chen collected clinical data. Tong Wu, Wei Liu, Jiajia Jin, and Suya Wang performed DNA extraction. Li Kuang designed the research study and reviewed the manuscript.

## Supporting information

Table S1‐S3
