# Supplementary material for: Targeted sequencing of the BDNF gene in young Chinese Han people with major depressive disorder
Source: Mol Genet Genomic Med. 2020 Aug 31;8(10):e1484. doi: 10.1002/mgg3.1484 (PMC7549566; doi:10.1002/mgg3.1484)
Supplement: Supplementary file 1 — Table S1‐S3 [file MGG3-8-e1484-s001.docx]

**Supplementary Material**

**Supplemental Tables**

**Table S1: Single nucleotide polymorphisms Information**

| SNP-ID | Position | Gene Region | Transcript  ID | Ref Allele | Alt Allele | Nucleotide change | MAF^a^ | MAF^b^ |
| --- | --- | --- | --- | --- | --- | --- | --- | --- |
| rs7124442 | 27677041 | UTR3 | NM_170735 | C | T | c.*2327G>A | 0.092 | 0.087 |
| rs11030099 | 27677583 | UTR3 | NM_170735 | C | A | c.*1785G>T | 0.492 | 0.425 |
| rs79642557 | 27678414 | UTR3 | NM_170735 | G | A | c.*954C>T | 0.084 | 0.100 |
| rs6265 | 27679916 | exonic | NM_170735 | C | T | c.196G>A | 0.484 | 0.448 |
| rs11030101 | 27680744 | UTR5 | NM_170735 | A | T | c.-633T>A | 0.25 | 0.289 |
| rs200712840 | 27681194 | UTR5 | NM_170735 | GTGCGCGCGCGC | - | c.1085_1083  delins- | NA | 0.107 |
| rs202011320 | 27681196 | UTR5 | NM_170735 | GCGCGCGCGC | - | c.1085_1085  delins- | NA | 0.113 |
| rs4030470 | 27681197 | Promoter | NM_170735 | C | T | NA | 0.478 | 0.110 |
| rs2883187 | 27741092 | UTR5 | NM_001143807 | G | A | c.-60981C>T | 0.399 | 0.454 |
| rs71050932 | 27742447 | Promoter | NM_170732 | - | CATTT | NA | 0.488 | 0.457 |
| rs3838785^*^ | 27679011 | UTR3 | NM_170735 | T | - | c.*357delA | 0.231 | 0.256 |

Ref: Reference;

Alt: Alternate;

MAF: minor allele frequency;

^a^: 1000 Genomes dataset (Chinese);

^b^: in control samples;

^*^: short tandem repeat

UTR: untranslated region;

NA: not applicable.

**Table S2: SNP-SNP Interaction Models, As Confirmed by GMDR**

| SNP-SNP interaction models | Training Balanced Accuracy | Testing Balanced Accuracy | Sign Test (P) | Cross-validation Consistency |
| --- | --- | --- | --- | --- |
| 1 | 0.5439 | 0.4635 | 3 (0.945) | 5/10 |
| 2 | 0.5776 | 0.5052 | 7 (0.171) | 7/10 |
| 3 | 0.6018 | 0.5063 | 5 (0.623) | 5/10 |
| 4 | 0.6236 | 0.5145 | 6 (0.377) | 5/10 |
| 5 | 0.6427 | 0.5451 | 7 (0.171) | 5/10 |
| 6 | 0.6496 | 0.5423 | 8 (0.054) | 5/10 |
| 7 | 0.6536 | 0.5143 | 7 (0.171) | 4/10 |

1: rs4030470;

2: rs79642557, rs6265;

3: rs79642557, rs6265, rs4030470;

4: rs79642557, rs6265, rs11030101, rs4030470;

5: rs7124442, rs79642557, rs6265, rs11030101, rs4030470;

6: rs7124442, rs79642557, rs6265, rs11030101, rs202011320, rs4030470;

7: rs7124442, rs11030099, rs79642557, rs6265, rs11030101, rs202011320, rs4030470;

**Table S3:** **Genotype and allele distributions of BDNF (rs3838785) short tandem repeat in young people**

| SNP-ID | Case (%) | | | Control (%) | | |  |  | MAF (%) | | χ^2^ | P-value | OR (95%CI) |
| --- | --- | --- | --- | --- | --- | --- | --- | --- | --- | --- | --- | --- | --- |
|  | M/M | M/m | m/m | M/M | M/m | m/m | χ^2^ | P-value | Case | Control |  |  |  |
| rs3838785 | 60.6 | 32.7 | 6.7 | 55.3 | 38.1 | 6.6 | 0.819 | 0.664 | 23.0 | 25.6 | 0.443 | 0.505 | 0.869(0.575-1.313) |

Case: young patients with major depressive disorder; Control: healthy young people; M: major allele; m: minor allele; MAF: minor allele frequency; OR: odds ratio; CI: confidence interval; NCBI Reference Sequence: NG_011794.1.
